# Joint association of polymorphism of the *FGFR4* gene and mutation *TP53* gene with bladder cancer prognosis

**DOI:** 10.1038/sj.bjc.6603456

**Published:** 2006-11-07

**Authors:** Y C Yang, M L Lu, J Y Rao, H Wallerand, L Cai, W Cao, A Pantuck, G Dalbagni, V Reuter, R A Figlin, A Belldegrun, C Cordon-Cardo, Z F Zhang

**Affiliations:** 1 Department of Epidemiology, UCLA School of Public Health and Jonsson Comprehensive Cancer Center, Los Angeles, CA 90095, USA; 2 Department of Pathology, Memorial Sloan-Kettering Cancer Center, New York, NY 10021, USA; 3 Department of Pathology and Laboratory Medicine, UCLA School of Medicine, Los Angeles, CA 90095, USA; 4 EMI INSERM 03-37 and Service d'Urologie, Université Paris XII, AP-HP, Hôpital Henri Mondor, 94000 Créteil, France; 5 Department of Epidemiology, Fujian Medical University, Fuzhou, Fujian, PR China; 6 Department of Urology, UCLA School of Medicine, Los Angeles, CA 90095, USA; 7 Department of Surgery, Memorial Sloan-Kettering Cancer Center, New York, NY 10021, USA; 8 Department of Medicine, Hematology-Oncology, UCLA School of Medicine, Los Angeles, CA 90095, USA

**Keywords:** bladder cancer, single nucleotide polymorphism, fibroblast growth factor receptor 4, prognosis, *TP53* mutation

## Abstract

The impact of the fibroblast growth factor receptor 4 (*FGFR4*) Gly388Arg polymorphism on bladder cancer is unknown. We found no clear correlations between the *FGFR4* genotype and risk of bladder cancer or pathological parameters. Neither the polymorphism nor *TP53* mutation status was an independent predictor of prognosis, but they might act jointly on the disease-specific survival of patients.

Bladder cancer is the fourth most common cancer in men and the ninth most common cancer in women in the United States. In 2006, there will be 61 420 new cases diagnosed and 13 060 will die from the disease (American Cancer Society, 2006). Owing to the highly heterogeneous clinical behaviors of bladder carcinoma, there is a need to stratify patients by molecular markers to optimise management and improve survival for patients with bladder cancer.

The fibroblast growth factor receptor (FGFR) family, which comprises of four structurally related tyrosine kinase receptors, transduces various crucial biological activities required for the growth and survival of cancer cells (Powers *et al*, 2000). Mutations in the *FGFR3* gene have been identified in 40–50% of bladder tumours, and were associated with a favourable prognosis with a lower recurrence rate and disease-specific mortality (Cappellen *et al*, 1999; Karoui *et al*, 2001; Sibley *et al*, 2001; van Rhijn *et al*, 2001). These mutations in the *FGFR3* gene are largely bladder cancer specific, and it is suggested that the FGFR family may play an important role in bladder carcinogenesis.

Recently, a common polymorphism in the transmembrane domain of the *FGFR4* gene, Gly388Arg, was identified by Bange's group (Bange *et al*, 2002). A positive correlation between the presence of the *FGFR4* Arg388 allele and prognostic parameters as well as survival time was reported in variant cancer studies, including breast, colon, lung, prostate, head and neck cancers and high-grade soft-tissue sarcoma (Bange *et al*, 2002; Morimoto *et al*, 2003; Streit *et al*, 2004; Wang *et al*, 2004; Spinola *et al*, 2005a). However, most studies were carried out with a relatively small sample size or suggested a possible effect only on a specific tumour type (Morimoto *et al*, 2003) or population (Wang *et al*, 2004). Furthermore, in subsequent larger studies of melanoma, breast, and colon cancer, no obvious association between the Gly388Arg genotype and cancer prognosis was found (Becker *et al*, 2003; Jezequel *et al*, 2004; Spinola *et al*, 2005b; Streit *et al*, 2006).

Mutations in the *FGFR3* and *TP53* genes are the two most frequent events observed in primary bladder tumours and have occurred in 59% and 25% of tumours, respectively (van Rhijn *et al*, 2004). *TP53* mutations are often found in advanced tumours and, thus, are associated with a poor prognosis. On the contrary, *FGFR3* mutations are associated with low-grade tumours and a favourable prognosis. This mutually exclusive expression suggests that *FGFR3* and *TP53* gene mutations may represent two alternative genetic pathways in the pathogenesis of bladder cancer.

To our knowledge, no study has been conducted on the prognostic significance of the *FGFR4* genotype in bladder cancer. Given the potential involvement of the FGFR family in bladder carcinogenesis, it is of interest to investigate the potential impact of the *FGFR4* Gly388Arg polymorphism and the combination of the *FGFR4* genotype and *TP53* mutation status on the progression of bladder cancer.

## MATERIALS AND METHODS

This study involved 140 newly diagnosed bladder cancer patients who underwent radical cystectomy and 151 healthy controls at the Memorial Sloan-Kettering Cancer Center from October 1993 to June 1997. All cancer patients were pathologically confirmed and staged according to the TNM staging system. After obtaining IRB-approved signed consents, all subjects were interviewed and had blood samples collected. Complete follow-up data were available for 140 cases with a median follow-up time of 33 months (range, 5–120 months). Genotyping of the *FGFR4* Gly388Arg polymorphism was determined with the RFLP analysis as described by Bange *et al* (2002). The status of *TP53* mutation was previously detected in the same samples by three essays, including manual sequencing, a commercial genechip-based assay (p53 Genechip) and immunohistochemistry (Lu *et al*, 2002). The survival time was calculated from the date of cystectomy to the date of disease-specific deaths, the date of recurrence or metastasis, or the date of the last follow-up. Survival curves were plotted according to the Kaplan–Meier estimate and compared using the long-rank test. The proportional hazards model was employed to estimate hazard ratios when adjusting for potential confounding factors. All statistic analyses were performed using SAS 8.1 software.

## RESULTS

The present study comprised of 140 bladder cancer patients and 151 healthy controls. Fifteen patients were excluded from the analysis because of poor quality DNA or indistinct genotyping results. One hundred and twenty-five patients who had similar distributions of demographic or pathological factors as the whole study group were included in the analyses. Among the 125 bladder cancer patients, 42.4% were heterozygous and 10.4% were homozygous carriers of the Arg388 allele. Compared with noncarriers, the OR of developing bladder cancer among Arg388 allele carriers was 1.05 (95% CI, 0.5–2.1) (data not shown). No obvious correlation was observed between the *FGFR4* Gly388Arg polymorphism and clinicopathological parameters (Table 1).

**Figure 1:**
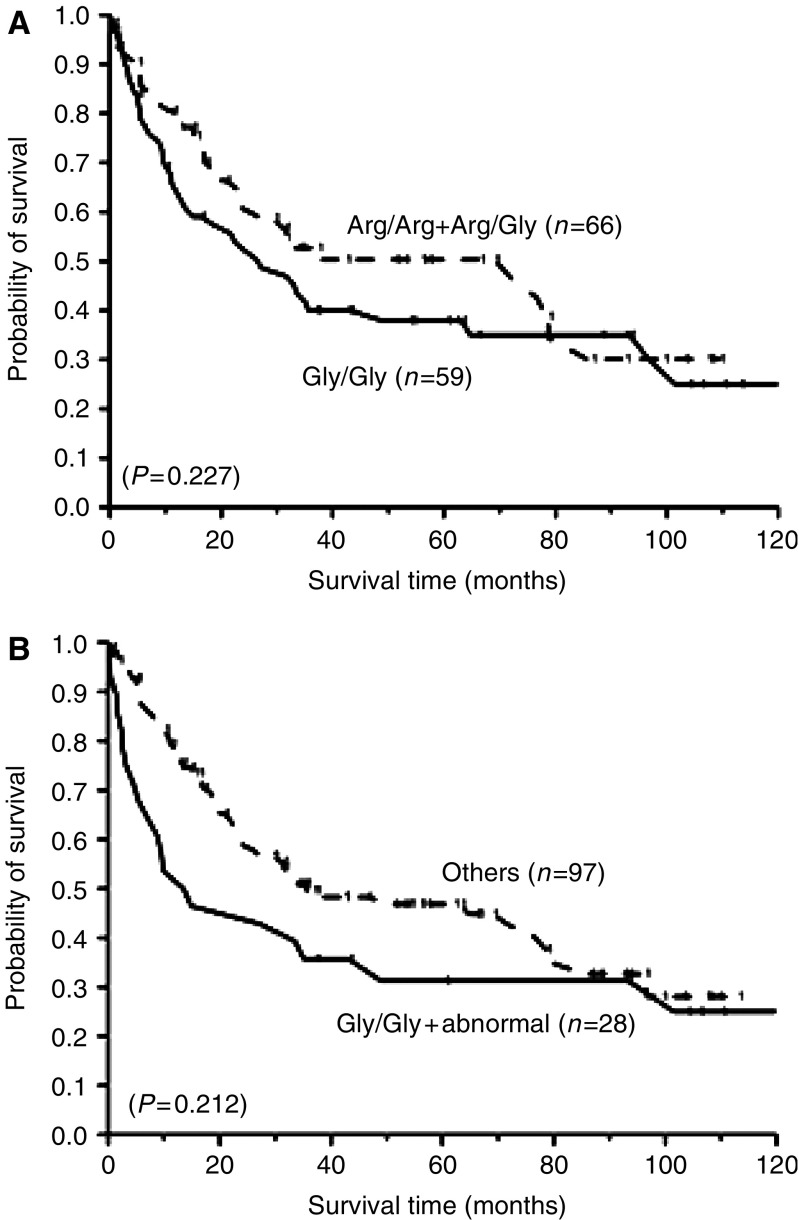
Kaplan–Meier disease-specific survival curves of bladder cancer patients. (**A**) Comparison between patients with *FGFR4* Gly/Gly genotype and patients with *FGFR4* Arg/Arg or Arg/Gly genotypes. The curves of Gly/Gly (*n*=59) and Arg allele carrier (*n*=66) patients are shown as solid and dashed lines, respectively. (**B**) Based on the combination of the *FGFR4* genotype and *TP53* mutations status, comparison between Gly allele homozygotes with abnormal p53 status (*n*=28, solid) and other (*n*=97, dashed). Small dots indicate censored observations. *P*-values were calculated by the log-rank test.

Of the 125 patients who were treated with radical cystectomy, 98 patients died during the 120-month follow-up period. Seventy three per cent of all deaths (*n*=72) were due to bladder cancer. No obvious difference was found between the *FGFR4* genotype or *TP53* mutations and the disease-specific survival (Table 2). The combination of both markers, however, was strongly associated with the disease-specific survival. *FGFR4* Gly388 homozygous patients with *TP53* mutation (median survival time=14.2 months) had a hazard ratio of 1.85 (95% CI, 1.01–3.39) for disease-specific survival compared with the reference group, which consisted of Gly388 homozygote without *TP53* mutation and Arg388 allele carriers with or without *TP53* mutation. (median survival time=23.3, 23.9 and 31.5 months, respectively) (Figure 1B).

**Figure 2:**
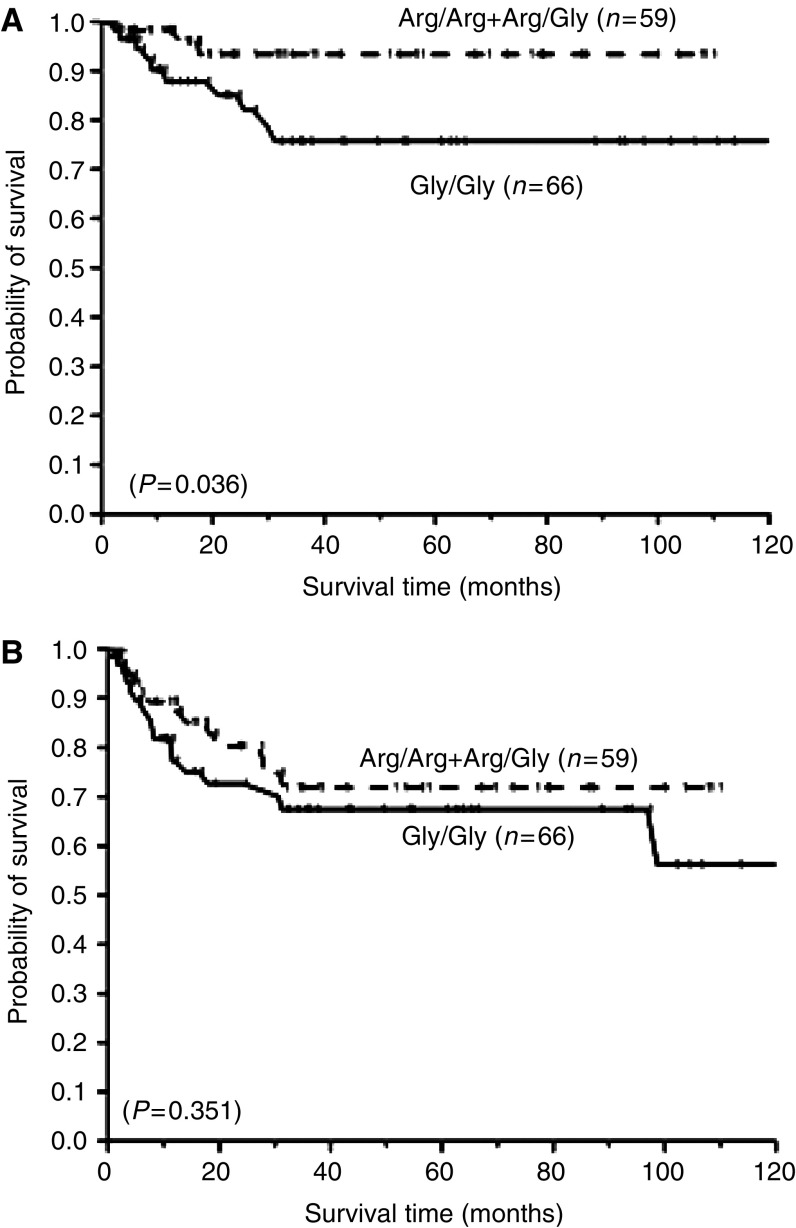
Survival of patients with bladder cancer according to variants of the *FGFR* Gly388Arg polymorphism. Log-rank analyses of the association between *FGFR4* genotypes and (**A**) recurrence-free survival time and (**B**) metastasis-free survival time in bladder cancer.

The *FGFR4* Gly/Gly genotype was associated with poor recurrence-free survival (*P*=0.036, Figure 2A), but not metastasis-free survival (*P*=0.351, Figure 2B). Due to the small proportion of patients who suffered the recurrence of bladder cancer during the follow-up period, we combined recurrence and metastasis and calculated the disease-free survival time for each case. After adjusting for age, gender and pathologic stage, patients with the Gly/Gly genotype or *TP53* mutation had a similar disease-free survival as Arg388 allele carriers (adjusted HR=0.99 and 1.01, respectively; data not shown).

## DISCUSSION

We observed no clear association between the Gly388Arg genotype and risk of bladder cancer, indicating that this SNP may not be involved in the early development of bladder cancer. Our finding is in agreement with several previous studies on other types of cancer (Bange *et al*, 2002; Morimoto *et al*, 2003; Spinola *et al*, 2005a), except for one study on prostate cancer (Wang *et al*, 2004).

The present study found no clear correlation between the *FGFR4* Gly388Arg polymorphism and pathological parameters. Furthermore, our results did not directly indicate that either the *FGFR4* genotype or *TP53* mutation status was an independent predictor of prognosis for bladder cancer but that they might act jointly on the disease-specific survival of patients, which supports the hypothesis that the *FGFR3* and *TP53* gene mutations may represent two alternative genetic pathways in the progression of bladder cancer (van Rhijn *et al*, 2004). A trend showed a better disease-specific survival rate for bladder cancer patients with the Arg/Arg genotype. This is contrary to the findings of previous studies on other types of cancer that the presence of the Arg allele is associated with poor survival (Bange *et al*, 2002; Morimoto *et al*, 2003; Streit *et al*, 2004; Wang *et al*, 2004; Spinola *et al*, 2005a). The reason for the conflicting results is unclear, but may reflect a tissue-specific effect of this polymorphism. For example, the activating somatic mutations in the *FGFR3* gene appear to be bladder specific, and a much lower frequency of the mutations of the *FGFR3* has been observed in other cancer sites. (Sibley *et al*, 2001; van Rhijn *et al*, 2001). The *FGFR4* Gly388Arg polymorphism results in an amino-acid change in the transmembrane domain, which is a highly conserved region for receptor tyrosine kinase. Analogous missense mutations in the transmembrane domain in the *FGFR3* gene, resulting from a Gly to Arg substitution at codon 380, were proposed to result in constitutive activation of *FGFR3* signaling (Webster and Donoghue, 1996). In general, activating *FGFR3* mutations are associated with favourable disease characteristics such as low stage and grade, low recurrence rate, and a lower mortality rate (Sibley *et al*, 2001; van Rhijn *et al*, 2001). Therefore, the *FGFR4* Gly388 allele, but not the Arg388 allele, could exert an effect on the aggressive behaviors of the cancer cells in bladder.

We also found that the patients with the Gly/Gly genotype were associated with a greater risk of recurrence, but were not associated with metastasis compared with Arg allele carriers. Our results are in accordance with a study of melanoma patients, which showed no correlation between the *FGFR4* genotype and metastasis (Streit *et al*, 2006). One possible reason for positive result in recurrence could possibly be due to false positives based on a relatively small number of recurrence cases (less than 10%).

Although the finding of differences in survival rates according to the combination of the *FGFR4* genotype and *TP53* mutation status is interesting, the results should be interpreted with caution. Because our study was limited to patients who underwent radical cystectomy, which is the standard treatment for muscle invasive bladder cancer (T2–T4), there are more patients with higher staging and grading tumours that have been recruited. However, no strong association between the *FGFR4* genotype and indicators for the aggressive tumour was found in our study population because of potential selection bias.

In summary, our results suggest that the variations in neoplastic progression not only depends on somatic mutations occurring in the tumour itself but also on the patient's genetic characteristics. However, the molecular mechanisms of the *FGFR4* Gly388Arg polymorphism has not been examined in human bladder cancer. Information on the function of this polymorphism or its potential biological interaction with TP53 is needed and may add information to optimise the treatment of patients with bladder cancer.

## Figures and Tables

**Table 1:** Associations between the *FGFR4* genotype and demographic characteristics, smoking status, pathological parameters, and *TP53* mutation status in 125 patients with bladder cancer

	**Gly/Gly, *n* (%)**	**Gly/Arg, *n* (%)**	**Arg/Arg, *n* (%)**	***P*-value**
All patients	59 (47.2)	53 (42.4)	13 (10.4)	
				
Age at diagnosis (years±s.d.)	67.0±9.6	64.7±10.9	69.7±9.5	0.220
				
*Age(years)*				
>66	35 (59.3)	29 (54.7)	8 (61.5)	0.868
≤66	24 (40.7)	24 (45.3)	5 (38.5)	
				
*Gender*				
Male	45 (76.3)	42 (79.3)	10 (76.9)	0.952
Female	14 (23.7)	11 (20.7)	3 (23.1)	
				
*Smoking status*				
Current	7 (13.2)	7 (14.6)	1 (7.7)	0.885
Former	36 (67.9)	32 (66.7)	8 (61.5)	
Never	10 (18.9)	9 (18.7)	4 (30.8)	
				
*Stage*				
I	4 (6.8)	3 (5.7)	1 (7.7)	0.993
II	5 (8.5)	5 (9.4)	1 (7.7)	
III	45 (76.2)	41 (77.4)	11 (84.6)	
IV	5 (8.5)	4 (7.5)	0 (0)	
				
*Grade*				
G1	3 (5.2)	3 (5.8)	0 (0)	0.924
G2	9 (15.5)	11 (21.1)	2 (15.4)	
G3-4	46 (79.3)	38 (73.1)	11 (54.6)	
				
*Lymph node involvement*				
Present	22 (37.3)	22 (41.5)	7 (53.8)	0.545
Absent	37 (62.7)	31 (58.5)	6 (46.2)	
				
*Vascular invasion*				
Yes	31 (52.5)	27 (50.9)	8 (61.5)	0.842
No	28 (47.5)	26 (49.1)	5 (38.5)	
				
*TP53 mutation*				
Yes	26 (44.1)	22 (41.5)	10 (76.9)	0.076
No	33 (55.9)	31 (58.5)	3 (23.1)	

**Table 2:** Hazard ratios of death from bladder cancer for the *FGFR4* Gly388 polymorphism and *TP53* mutation status in 125 cancer patients

	**No.**	**Death**	**Death (%)**	**Hazard ratio^a^**	**95% CI**	***P*-value**
*FGFR4 genotype*						
Gly/Gly	59	38	64	1.42	(0.83–2.40)	0.198
Arg/Arg+Arg/Gly	66	34	52	ref.		
						
*TP53 status*						
Abnormal	62	38	61	1.53	(0.88–2.65)	0.133
Normal	63	34	54	ref.		
						
*FGFR4/TP53*						
Gly/Gly/abnormal	28	21	75	2.25	(1.04–4.88)	0.039
Gly/Gly/normal	31	17	55	1.28	(0.58–2.83)	0.538
Arg/Arg+Arg/Gly/ abnormal	34	17	50	1.38	(0.63–3.04)	0.423
Arg/Arg+Arg/Gly/ normal	32	17	53	ref.		
						
Gly/Gly+abnormal	28	21	75	1.85	(1.01–3.39)	0.046
Other	97	51	53	ref.		

a Hazard ratios were adjusted for age, gender, lymph node involvement, tumour stage and grade.
